# Indigenous and Invasive Fruit Fly Diversity along an Altitudinal Transect in Eastern Central Tanzania

**DOI:** 10.1673/031.012.1201

**Published:** 2012-01-27

**Authors:** Katrien Geurts, Maulid Mwatawala, Marc De Meyer

**Affiliations:** ^1^ Terrestrial Ecology Unit, Faculty of Sciences, Ghent University, Gent, Belgium; ^2^ Department of Crop Science and Production, Sokoine University of Agriculture, Morogoro, Tanzania; ^3^ Entomology Section, Royal Museum for Central Africa, Tervuren, Belgium

**Keywords:** *Ceratitis*, *Bactrocera*, inter-specific competition, climate change

## Abstract

The relative abundance of indigenous and invasive frugivorous fruit flies (Diptera: Tephritidae) was evaluated spatially and temporally along an altitudinal transect between 581–1650 m in the Uluguru Mountains near Morogoro, Tanzania. The polyphagous invasive fruit fly *Bactrocera invadens* Drew, Tsuruta, and White and the indigenous fruit fly *Ceratitis rosa* Karsch show a similar temporal pattern, but are largely separated spatially, with *B. invadens* being abundant at lower elevation and *C. rosa* predominant at higher elevation. The polyphagous indigenous *C. cosyra* (Walker) coincides with *B. invadens* but shows an inverse temporal pattern. The cucurbit feeders *B. cucurbitae* (Coquillett) and *Dacus bivittatus* (Bigot) show a similar temporal pattern, but the former is restricted to lower elevations. Host availability and climatic differences seem to be the determining factors to explain the differences in occurrence and abundance in time and space.

## Introduction

The increasing number of invasive species is one of the greatest threats to global diversity (Dukes and Mooney 1999) as well as to ecology, evolution (Sakai et al. 2001), and local economies (Sax et al. 2007). Ecologically diverse tropical regions in developing countries are especially vulnerable because of their often-precarious economic systems and security programs, and the limited financial means at their disposal to monitor and control unwanted invasions. A successful invasion depends on transport, colonization, establishment, and spread of the pest (Sakai et al. 2001). Successful completion of each of these stages depends on filters at work on different scales, including climate change (Theoharides and Dukes 2007). Dukes and Mooney (1999) emphasize the significant effect the latter can have on current and future invasions and the need for the incorporation of this element in invasion models. Altitudinal gradients can serve as spatial analogues for climate change (Bale et al. 2002). Like climate change, ecological gradients such as host plants and predators (Vayssières et al. 2008), as well as physical gradients like temperature, rainfall, and humidity (Hodkinson 2005) encountered along an altitudinal transect can have an impact on the density, diversity, and life history of insects and demands for phenotypic flexibility and genotypic adaptability of many species (Bale et al. 2002).

Fruit flies (Diptera: Tephritidae) are a good model taxon for ecological research on invasion success. Many fruit fly species have high reproductive rates and good (passive) dispersive powers amplified by not only the current increase in global trade of fruit products but also by an increasing number of tourists who may carry infested fruits (Liebhold et al. 2006). These characteristics make fruit flies excellent invaders (Ward and Masters 2007) and allow them to become pests in certain places (Ekesi et al. 2007).

*Bactrocera cucurbitae* (Coquillett), a fruit fly from Asia, became an invasive pest species in Africa after first establishing itself in Eastern Africa and appearing in Western Africa at the beginning of this century (Vayssières et al. 2008; Virgilio et al. 2010). It is considered a major pest of several Cucurbitaceae, while occasionally infesting non-cucurbit fruits (Mwatawala et al. 2010). Another major Asian invasive species, *B. invadens* Drew, Tsuruta, and White, was first detected in Kenya in February 2003 followed by Tanzania (Mwatawala et al. 2004), and has now been recorded in 27 African countries (Rwomushana et al. 2008b; De Meyer et al. 2010). Currently, its spread appears to be continuing not only in latitude but also in altitude. In Kenya and Tanzania, the highest fruit infestation rates are recorded at lower altitudes (Ekesi et al. 2006; Rwomushana et al. 2008a; Mwatawala et al. 2009a). Recently, however, mango infestation has also been recorded at medium to high altitudes in the Eastern Province of Kenya (Rwomushana et al. 2008a), which was not the case in previous studies (Ekesi et al. 2006). In Tanzania *B. invadens* occurs most prominently at low and medium altitudes (< 1600 m) (Mwatawala et al. 2006a), but individuals have been detected at higher altitudes (Mwatawala et al. 2006b).

*Bacterocera invadens* spread and successful colonization of higher altitudes could be limited by climatic conditions (Mwatawala et al. 2006a), host availability and suitability (Rwomushana et al. 2008a), and interspecific competition with cold-tolerant species like *Ceratitis rosa* Karsch (Mwatawala et al. 2006a). Temperature is usually considered to be the most important factor explaining population dynamics in insect species (Vayssières et al. 2008), as well as humidity to some extent (Duyck et al. 2004). Extreme variations such as drought and inundation are the greatest threats to the most vulnerable stage of fruit fly life history: the pupal stage (Duyck et al. 2004; Duyck et al. 2006b).

In addition to invasive *Bactrocera* species, several indigenous fruit fly pests occur in Eastern Central Tanzania, especially belonging to the Afrotropical genus *Ceratitis* (Mwatawala et al. 2006a). The genus *Bactrocera* is renowned for its strong competitive abilities and capacity to largely displace other (indigenous) fruit fly species (Duyck et al. 2006a, 2006b). Species composition and density along altitudinal gradients change according to the niche width of different species (Hodkinson 2005). In this paper we investigate species composition of fruit flies spatially and temporally in relation to environmental gradients along an altitudinal transect in Eastern Central Tanzania.

**Figure 1.**
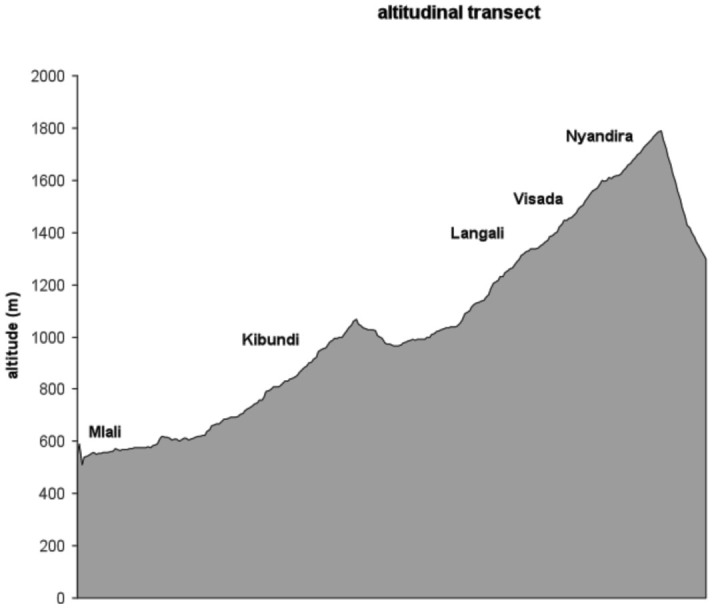
Altitudinal distribution of sampling stations. High quality figures are available online.

## Materials and Methods

### Study site

Studies were conducted in the Morogoro Region of Tanzania (05° 58′ S - 10° 00′ S, 35° 25′ E - 38° 30′ E) from September 2008 to September 2009 (Table 1). Located in Eastern Central Tanzania, Morogoro has a subtropical climate and is situated in the transition zone between the bimodal and unimodal rainfall belts of Tanzania. There are two rainy seasons: one short (November–December) and the other long (March–May). The annual rainfall varies between 750–1400 mm and can fluctuate considerably between years.

The transect (Figure 1) was placed in an embranchment of the Uluguru Mountains, which is part of the Eastern Arc Mountains. The vegetation consisted of cultivated land, including plantations of maize, sugar cane, beans, and fruit orchards (mango (*Mangifera indica*), citrus (*Citrus* sp.), peach (*Prunus persica*), apple (*Malus domestica*), jambolan (*Syzigium cumini*), avocado (*Persea americana*), papaya (*Carica papaya*), feijoa (*Feijoa sellowiana*), and guava (*Psidium guajava*)), as well as fallow land overgrown with grasses and shrubs. Peach, apple, and pear are temperate fruits and according to their climatic demands they can only be grown at high altitudes. Most tropical fruits do not occur above a certain altitudinal limit for the same reason. The crops are cultivated by terracing and are grown in polycultures.

### Traps

Modified McPhail® traps (Scentry Biologicals Inc., www.scentry.com) were set at five stations along the transect (500–1650 m) at similar intervals (Table 1, Figure 1). Traps were hung on fruit trees, usually mango, except at the high altitude sites where traps were also hung in peach (Visada and Nyandira), plum (Nyandira), and apple (Nyandira) trees. They were baited with one of four different parapheromones (methyl eugenol (ME), cue lure (CL), terpinyl acetate (TA) and trimedlure (TM)), each attracting a different part of the fruit fly diversity in the region (Mwatawala et al. 2006a). Where required, sticky glue was applied on the branches to prevent ant predation. At every trapping station there were three replicate sets of traps. Each set consisted of four traps each with a different lure and a killing agent dichlorovos (vapona). The traps were active (lure and insecticide added) one out of every four weeks. One week after activation the traps were emptied, randomized across the tree, and de-activated (lure and insecticide removed). The captured flies for each sample were uniquely coded and brought to the lab where the specimens were counted, identified, and finally preserved in 70% ethanol. At each site an iButton® data logger (Maxim Integrated Products, www.maxim-ic.com) was placed that recorded temperature (resolution 0.5 ^°^C) and RH (resolution 0.6%) every two hours. Daily and monthly averages of these measurements were calculated and used to interpret the results.

At each trapping station, fruit was collected every two weeks for rearing experiments (results not discussed in this paper); the fruit species collected every month were used as an indication of host presence along the transect (data shown in Appendix 1). Selection of fruit species was based on earlier rearing experiments (Mwatawala et al. 2009b).

### Statistical analysis

The influence of different environmental factors on species composition and abundance was explored using ordination techniques, correlation analyses, and Kruskal-Wallis tests. Temperature, humidity, and host presence (of mango, peach, and soursop) were the environmental factors used in this study. Ordination was carried out using Canoco 4.5 (ter Braak and Šmilauer 2002). Relative abundance of every species along the transect was used. The species data were square-root transformed and rare species were down weighted. The significance of every variable in the Canonical Correspondence Analysis (CCA) or Redundancy analysis (RDA) was tested using a Monte Carlo permutation (499 permutations). In addition, a series of constrained, detrended CCA (DCCA) were run with the species data and each environmental variable on its own. This was to determine the strength of each variable by its ability to maximize the dispersion of the species scores, expressed as a ratio of the eigenvalue of the first axis to the eigenvalue of the second axis (Zhang et al. 2007; Woodward and Schulmeister 2008). A low value could point out a factor not considered. The unique explanatory power of each significant environmental variable was tested using a series of partial, constrained ordinations (RDA or CCA) with all other significant environmental parameters as covariables (Woodward and Schulmeister 2008). The ordination analyses were an exploratory method to filter out the most important environmental variables. To study their influence in more detail the data were analyzed using correlation techniques. The data did not have a normal distribution, variances were not homogenous, and there was a correlation of the mean and the variance; thus, non-parametric tests needed to be used. Spearman's rank correlation analyses and Kruskal-Wallis tests were performed in R 2.12.2 (R Development Core Team 2008). For Spearman's rank correlations absolute abundances were used, and for Kruskal-Wallis tests proportional abundance of a species at a certain time at a certain site were square-root arcsin transformed. Proportional abundance was used to avoid effects of attractiveness of lures.

**Figure 2.**
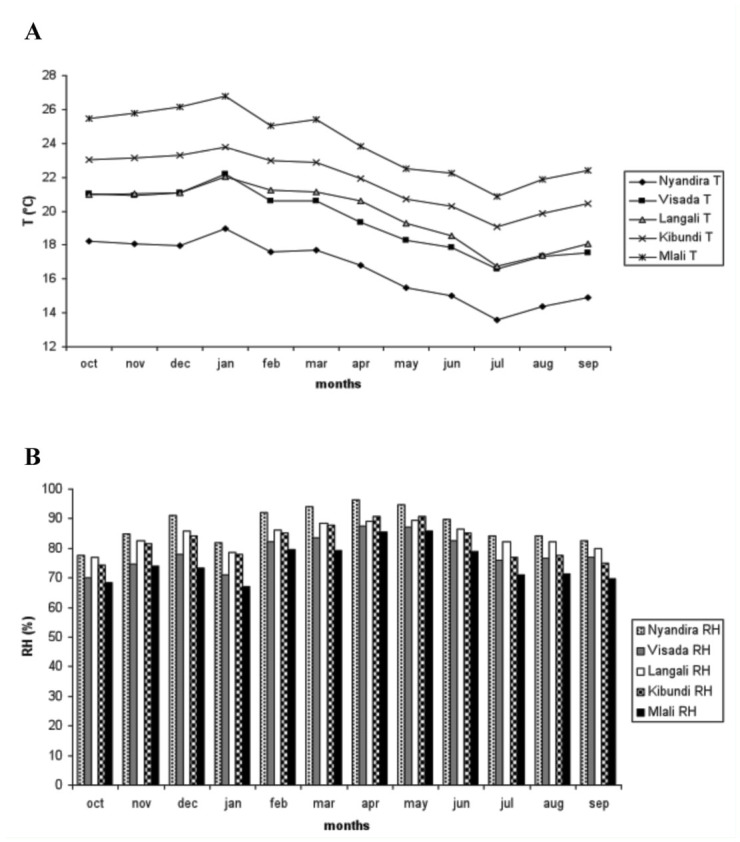
(a) Temperature and (b) RH at different elevations over time. High quality figures are available online.

## Results

### Temperature and relative humidity variation along the transect

Throughout the year, the temperature was lower at sampling sites situated at higher elevations (Figure 2a). The monthly average difference between the highest and lowest sampling point was consistently between 7–8 ^°^C and there was a clear gradient except between Visada and Langali, which have largely similar temperatures. On average, the higher sampling sites showed a higher RH compared to the lower sites (Figure 2b). Again, Visada (the second highest sampling point) was an exception, with a lower RH than the third highest (Langali) and often the fourth highest (Kibundi) sites. This is because Visada is a sun-exposed site and therefore captures a lot of heat and loses humidity. The months October to January experienced a high temperature and low RH (Figure 2), with the highest values in January. The months February (just before the start of the long rainy season) to April (peak of the long rainy season), demonstrated an intermediate temperature and high RH, with the highest RH in April. Both factors declined towards and during the dry season with the lowest temperature and a low RH in July. In 2008, the short rainy season was largely absent.

### Temporal and spatial differences

**Figure 3.**
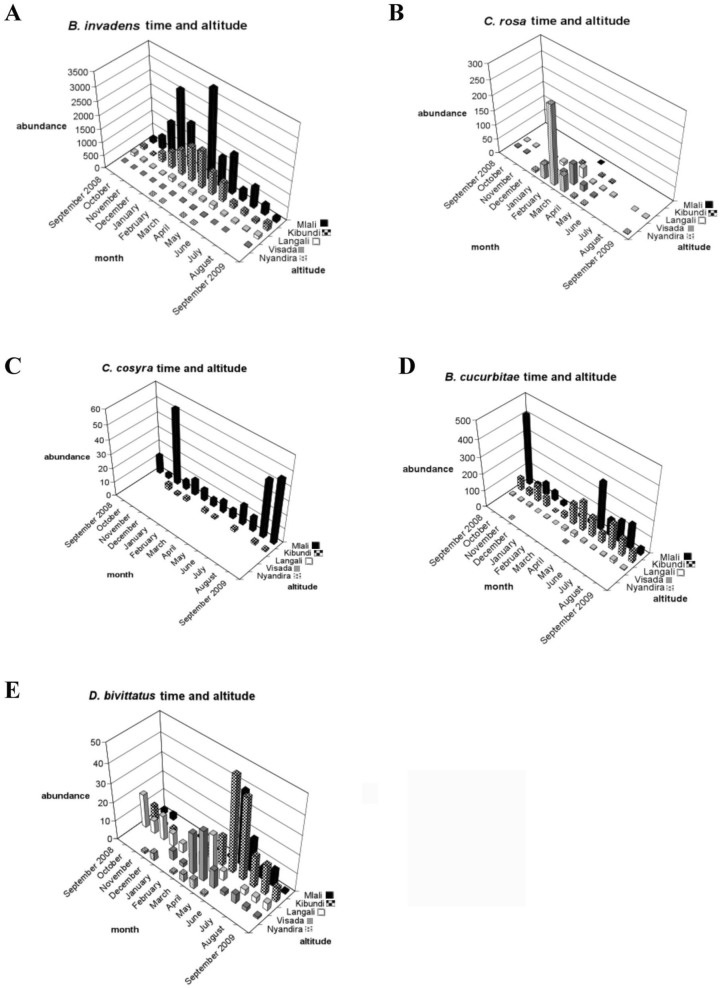
Temporal and altitudinal distribution of five dominant fruit fly species: (a) *Bactrocera invadens*, (b) *Ceratitis rosa*, (c) *C. cosyra*, (d) *B. cucurbitae*, and (e) *Dacus bivittatus.* High quality figures are available online.

In total, 16 different species were recorded (Appendix 2). Five species were predominant in the trap catches: *B. cucurbitae, B. invadens, C. cosyra* (Walker), *C. rosa*, and *D. bivittatus* (Bigot). The temporal and spatial occurrence for these is shown in Figure 3. The temporal abundance observed throughout the different sites for the dominant species showed two distinct patterns: *B. invadens* and *C. rosa* showed a peak period between December (*B. invadens*) or January and March (*C. rosa*) (Figure 3a, 3b). *Ceratitis cosyra* showed an inverse pattern with the highest abundance between August and November (Figure 3c). There was a significant difference in total fruit fly abundances between months (*p* < 0.01). Significant differences between months in fruit fly abundance were found in the months May to November. Studying every fruit fly species individually showed that for *C. rosa* and *D. bivittatus* there was a significant difference in abundances between months (Table 2). Differences for dominant species are summarized in Table 3. For *B. invadens* there were no significant differences between sites within months. For *B. cucurbitae* significant differences between sites occurred in March, April, May, June, July, August, and September. For *C. rosa* significant differences between sites occurred in January, February, March, April, and June. For *C. cosyra* significant differences between sites within months occurred in January, February, April, June, and July. For *D. bivittatus* significant differences between sites occurred in June.

Looking at spatial patterns for dominant fruit fly species showed that the abundance of some fruit fly species (*B. invadens, B. cucurbitae, and C. cosyra*) declined with rising altitude, while that of other species (*C. rosa* and *D. bivittatus*) increased (Figure 3). There was a significant difference in total fruit fly abundances between sites (*p* < 0.01). Significant differences between sites were found in Nyandira and Visada. Studying every fruit fly species individually showed that there was a significant difference between sites for each fruit fly species (Table 4). Regarding the dominant species, *B. invadens* (Table 5), there were significant differences between months in Nyandira. For *B. cucurbitae* and *C. cosyra* there were no significant differences between months within sites. For *C. rosa* there were significant differences between months in Langali, Visada, and Nyandira. For *D. bivittatus* there were significant differences between months in Kibundi and Visada.

### Ordination analysis of species and environmental variables

From the DCA-analysis it was clear that the first axis explained most of the variation (λ1 = 0.729). The length of the gradient was 5.263, which indicates a unimodal species response model and thus a CCA was used for further analysis of these data.

The CCA showed that the first two axes (axis 1 = 0.460; axis 2 = 0.186) explained 22.3% of the variation of the species composition of the trapped fruit flies (Table 6). The ratio of the eigenvalues of the axes was relatively high (2.473), which indicates that most of the variation was explained by the explored environmental variables. The species-environment correlations for axis 1 and axis 2 were 0.931 and 0.603, respectively. These two axes explained 65.4% of the variation of the relationship between fruit flies and environmental variables, which suggests a strong relationship. The variables temperature (T) and *Prunus persica* (Pp) had the strongest correlation with the first axis (T = -0.6269, Pp = 0.5196); this pattern held up even when the variables were analyzed separately (see Table 6). Examining the partial CCAs more in detail showed that T explained most of the variation in species composition (8.5%), followed by Pp, which explained 6.5% of the variation. These results were significant at a 5% significance level. Other variables that explained a significant proportion of the variation were RH (4.5%) and *Annona muricata* (Am) (3.7%). When all environmental variables that explained most of the variation were analyzed again with all the other variables as covariables, only temperature explained a significant proportion of the variation, independent of all other environmental variables (Table 6).

**Figure 4.**
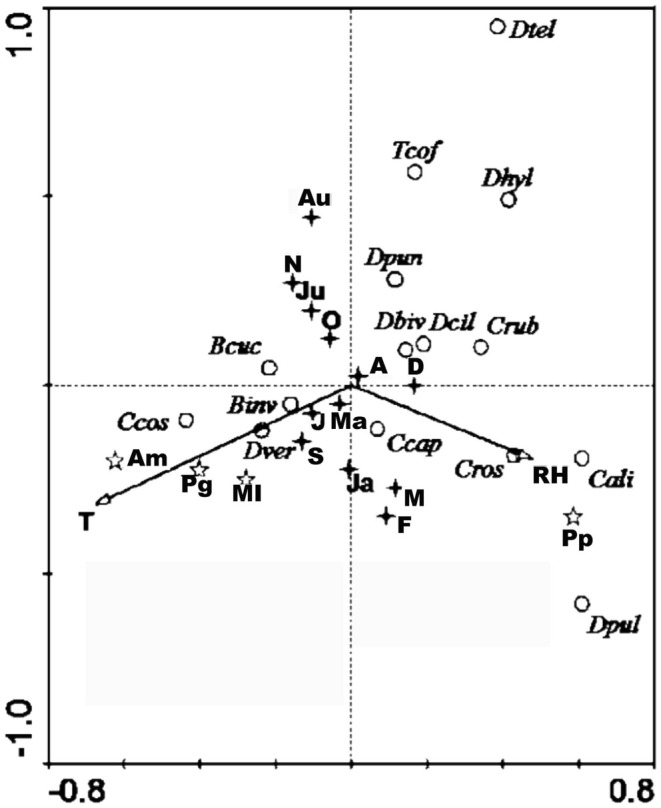
Biplot of CCA analysis. October (O), November (N), December (D), January (Ja), February (F), March (M), April (A), May (Ma), June (J), July (Ju), August (Au), September (S), *Annona muricata* (Am), *Psidium guajava* (Pg), *Prunus persica* (Pp), *Mangifera indica* (Mi), temperature (T), relative humidity (R). High quality figures are available online.

The biplots of the CCA illustrate the patterns more in detail (Figure 4). The biplot modeling species and environmental variables (Figure 4) showed that *B. invadens, C. cosyra*, and *B. cucurbitae* are most correlated with a high temperature (T), a low relative humidity (RH), and the presence of mango (Mi), soursop (Am), and guava (Pg). *Ceratitis cosyra* had a closer association with soursop than *B. invadens.*

*Ceratitis rosa* and *D. bivittatus* were most associated with a lower temperature and a higher RH. *Ceratitis rosa* was most correlated with the presence of peach (Pp).

### Correlation analysis of dominant species

The overall abundance of *B. invadens, B. cucurbitae*, and *C. cosyra* were significantly positively correlated with temperature, while those of *C. rosa* and *D. bivittatus* were not (Table 7). When studying the abundance of *B. invadens* in detail at every site, its abundance was significantly positively correlated with temperature only at Mlali (r = 0.68, *p* < 0.05). For *B. cucurbitae* a similar correlation was found at Kibundi (r = 0.59, *p* < 0.05), and for *C. rosa* at Langali (r = 0.68, *p* < 0.01). There was no significant correlation between the abundance of *C. cosyra* and temperature in any of the sites considered. For *D. bivittatus*, there was only a significant negative correlation at two sites (Kibundi: r = -0.82, *p* < 0.01; Mlali: r = -0.66, *p* < 0.05).

The overall abundance of *B. invadens, B. cucurbitae*, and *D. bivittatus* were not significantly correlated with RH, while that of *C. rosa* was highly significantly positively correlated and that of *C. cosyra* was significantly negatively correlated with RH (Table 8). When studying the abundance of *B. invadens* in detail at every site, its abundance was significantly positively correlated with RH only at Kibundi (r = 0.71, *p* < 0.01). For *B. cucurbitae* there was no significant correlation at any of the sites. For *C. rosa*, a significant positive correlation was found only at Kibundi (r = 0.67, *p* < 0.05). There was no significant correlation between the abundance of *C. cosyra* and RH in any of the sites considered. For *D. bivittatus* a significant positive correlation was found only in Nyandira and Visada (Nyandira: r = 0.83, *p* < 0.01; Visada: r = 0.61, *p* < 0.05).

## Discussion

*Bactrocera invadens, C. rosa*, and *C. cosyra* are reported from a wide variety of hosts throughout Africa (De Meyer et al. 2002; De Meyer et al. 2008), as well as in this particular study area (Mwatawala et al. 2009b). *Bactrocera cucurbitae* is an oligophagous species attacking a wide variety of wild and commercially grown Cucurbitaceae, while *D. bivittatus* seems to have a slightly more restricted cucurbit host range in the study area (Mwatawala et al. 2010). Caution should be taken when comparing the trapping results obtained through different attractants, since the relative effectiveness of the different parapheromones used differs widely (see for example Shelly et al. 2010; Vargas et al. 2010). Therefore, proportional abundance was used to correct for any lure attractiveness effect. However, the results obtained allow a comparison for each individual species in time and space since the effectiveness of a particular lure is not affected by these factors.

The temporal differences in overall proportional abundances were only significant for *C. rosa* and *D. bivittatus* (Table 2). During the wet months (February and March) *C. rosa* occurred in high abundance in higher altitude areas (Nyandira and Visada) (Figure 3b). This illustrates this fruit fly's preference for periods with a high relative humidity, which can also be deducted from its positive correlation with RH (Table 8). It also only occurs in Kibundi during these months with a high RH (Figure 3b). The distinct peak in February and March in Nyandira, Visada, and Langali corresponds with ideal conditions for this fruit fly: low temperature, high RH, and fruiting of one of its preferred hosts, peach (Figure 3b). This pattern can also be seen on the biplot of the ordination analysis (Figure 4). *Dacus bivittatus* occurred in periods with a high RH and low temperature. *Dacus bivittatus* abundance had a positive correlation with RH in high altitude areas (Table 8), illustrated by peaks in abundance in March and April in Visada (Figure 3e), and a negative correlation with temperature in low lying areas (Table 7), illustrated by its peak in abundance in May and June in Kibundi (Figure 3e).

For *B. invadens*, significant differences between months were found in Nyandira, where it was present in January, March, and April, and absent during the other months (Figure 3a). The abundance of *B. invadens* was positively correlated with temperature (Table 7) and its presence in Nyandira during these warmer months illustrates this. This can also be explained by a host effect. Earlier host rearing experiments (Mwatawala et al. 2006b, 2009b) have shown that *B. invadens* has potential hosts available throughout the year, but that one of its preferential hosts, mango, is available from November to February, and that fly populations show a distinct peak coinciding with this fruit, as also seen during this study (Figure 3a). From the ordination analysis it was shown that mango explained a large portion of fruit fly relative abundance variation (Table 6), and that mango was a determining factor for *B. invadens* (Figure 4). However, mango trees are only present along the altitudinal transect up to 1302 m, and in higher altitude areas alternative hosts such as peach guarantee fruit fly reproduction. Peach becomes available as a food source during February and March, which explains the rise in *B. invadens* abundance in March and April at higher altitudes. A second peak in March–May at the lower altitudes, coinciding with the guava season, is only marginally present. A large variety of alternative hosts fruiting at different periods in the year clarify the presence of *B. invadens* throughout the year and the absence of significant differences in abundance between months.

The seasonal abundance observed for *B. cucurbitae* also largely corresponds with earlier findings in Morogoro (Mwatawala et al. 2010), where a peak period was observed between May and October (Figure 3d), coinciding with the availability of cucurbit hosts (although *B. cucurbitae* is known to attack non-cucurbits, these infestations are largely occasional and of low significance). These peaks in abundance were also present during environmental conditions that seem to be preferred by this fruit fly species—periods with intermediate to high temperature and intermediate RH.

*Ceratitis cosyra* showed a positive correlation with temperature (Table 7) and a negative correlation with RH (Table 8), illustrated by its high abundance during dry and relatively warm months in Mlali (November, August, and September 2009) (Figure 3c). These peaks in abundance can also be due to a host effect.

The biplot of the ordination analysis shows a close association of *C. cosyra* with mango, but an even closer association with soursop (Figure 4a). In addition to mango, the preferential host range for *C. cosyra* is Annonaceae. A study from 2004–2006 detected a host shift by *C. cosyra* at the end of the mango season (April/May) towards soursop (*A. muricata*) (Mwatawala et al. 2009b). In our study, *C. cosyra* showed a peak in November, corresponding with the early mango season, and a peak in August and September, when soursop is available but mango is not (Figure 3c).

The temporal occurrence for all these species seems to be defined by preferred host availability and environmental conditions. This could also be concluded from the ordination analysis. Temperature and peach explained most of the variation in species composition (Table 6). RH and soursop explained another significant portion of the variation (Table 6). When considering all the other variables as covariables, only temperature still had significant explanatory power (Table 6). The fact that the fruits no longer explained significant portions of the variation is because environmental conditions determine phenology and nutrient quality of hosts (Hodkinson 2005). By separating out the effects it was revealed that underlying the availability of hosts, temperature is the most important factor in explaining fruit fly abundance, which has been concluded from several previous studies (Vargas et al. 1997; Bale et al. 2002; Vayssières 2008). Although RH was no longer a significant factor when considered on its own, it still explained a large part of the variation (Table 6). Rainfall is considered to be the most important factor for growth and quality of host plants (Mwatawala et al. 2006b). At the end of 2008, the short rainy season was delayed, which caused mangoes to ripen later in the season and to be smaller in size.

The spatial patterns can be explained by temperature, RH, and host differences between the sites. *Bactrocera invadens* and *B. cucurbitae* showed a positive correlation with temperature (Table 7), and therefore they occur in highest numbers in low-lying areas. *Bactrocera invadens* spatial occurrence observed here confirms earlier findings (Mwatawala et al. 2006a), showing that its presence declines at higher elevations (Figure 3a). Although suitable hosts such as mango still occur at the higher elevations, the abundance is very low. De Meyer et al. (2010) showed in their climatic modeling that the most suitable areas for establishment of *B. invadens* corresponded with the Equatorial climate category of the Köppen-Geiger climate classification (Kottek et al. 2006), which is categorized by minimum average temperature at or above 18 ^°^C. These conditions are mainly found at the two lowest sites (Mlali and Kibundi). *Bactrocera invadens* sporadic occurrence at high altitudes (above 1600 m), however, indicates that this fruit fly species has a large invasive potential and may be spreading and/or adapting to higher altitudes with lower temperatures.

*Ceratitis cosyra* had a positive correlation with temperature (Table 7) and a negative correlation with RH (Table 8), and therefore seems to be even more restricted to lower areas. No climatic model has been developed for *C. cosyra*, but at our study site, it was only relatively abundant at the lowest sampling site. The abundance of *C. cosyra* in Benin was observed to be high in dry periods and quickly dropped when the rains started (Vayssieres et al. 2005, 2009), corresponding with our findings. In Kenya, however, *C. cosyra* was recorded in higher altitudinal areas (Ekesi et al. 2006), which was not found in our study.

*Ceratitis rosa* showed a positive correlation with RH (Table 8), and for the ordination analysis was associated more with low temperature areas (Fig 4a); therefore, it occurred more often in high altitude areas. Previous studies confirm that *C. rosa* is a species that can withstand colder temperatures (Duyck et al. 2004, 2006b; De Meyer et al. 2010), although it is has also been reported in warm and wet conditions (Grout and Stoltz 2007). *Ceratitis rosa* also showed a close association with peach (Figure 4), which is only found at higher altitudes (Visada and Nyandira), explaining its presence there in high abundance.

*Dacus bivittatus* abundance had a positive correlation with RH in high altitude areas, and a negative correlation with temperature in low-lying areas. Therefore, it can occur in high abundances in areas with intermediate temperatures and RH such as Langali and Visada.

Survival of fruit flies with a narrow temperature and host range is therefore not possible along the entire altitudinal range. For these reasons, spatial shifts from low altitude to the highest altitude seem very unlikely. However, it is possible that the spatial shift of species is in an intermediate phase. Visada should be the ideal location for this phenomenon, as it has hosts from high (e.g., peach) and low (e.g., mango) altitude areas and has an intermediate temperature and humidity range. Therefore, survival of all fruit fly species is possible in this location, and thus host shifts and adaptation to lower and/or higher temperatures could occur here.

In general, the presence of suitable hosts and environmental conditions seem to be decisive for abundance patterns and species composition along the transect. The temporal and spatial patterns observed during this one-year study need to be substantiated by repeated year cycles, but are already supported to some extent through other studies conducted in the study area (Mwatawala et al. 2006a, 2006b, 2009b, 2010). Although *B. invadens* does not occur permanently at high altitude, future colonization is possible. Every year in January and February, this fruit fly appears to be dispersing towards higher areas (Mwatawala et al. 2006a), especially at times when there is a population surplus. Both the rapid population increase (indicating r-selection) (Duyck et al. 2007) and the broad host range (Mwatawala et al. 2006b) are ideal characteristics to allow for further spread and colonization of new areas. The effect that climate change may have is at this stage hard to predict. However, recent studies indicate warming at higher elevations in the tropics (Eggermont et al. 2010; Chen et al. 2009), which could lead to a more suitable environmental space at high altitudes for this invasive species. In particular, the impact of shifting minimal temperature thresholds on fruit fly distribution because of climate change has been demonstrated in *Rhagoletis* (Aluja et al. 2011), and a similar pattern might have an impact on the altitudinal move of *B. invadens.*

**Table 1.**
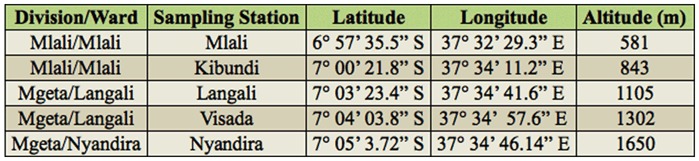
Geographical position of trapping stations.

**Table 2.**
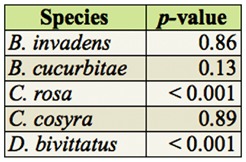
Temporal differences between months for each fruit fly species using Kruskal-Wallis tests.

**Table 3.**
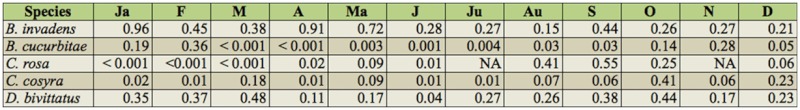
*p*-values for Kruskal-Wallis tests for each fruit fly species for differences between sites within months (NA: no individuals caught).

**Table 4.**
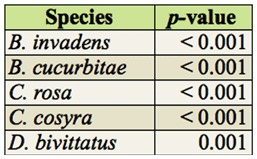
Spatial differences between trapping stations for each fruit fly species using Kruskal-Wallis tests.

**Table 5.**
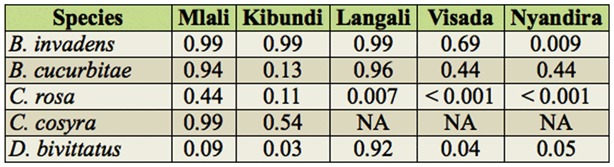
*p*-values for Kruskal-Wallis tests for each fruit fly species for differences between months within sites (NA: no individuals caught).

**Table 6.**
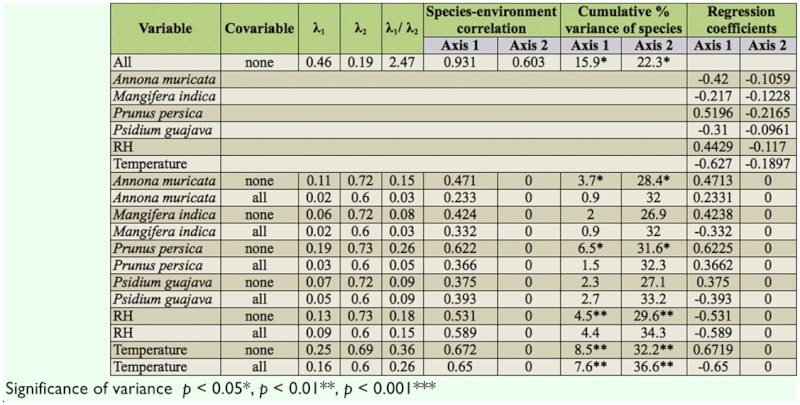
CCA (only environmental values reported).

**Table 7.**
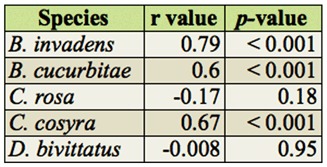
Spearman's rank correlation of overall abundance dominant fruit fly species and temperature.

**Table 8.**
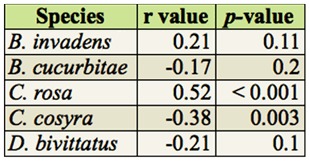
Spearman's rank correlation of overall abundance dominant fruit fly species and RH.

## Figures and Tables

**Table:**
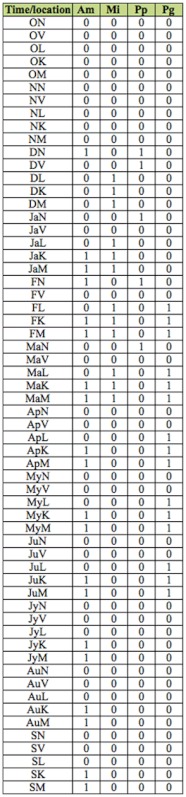
Host presence (1)/absence (0) for different Locations: Kibundi (K), Mlali (M), Nyandira (N), Visada (V), Langali (L) Months: October (O), November (N), December (D), January (Ja), February (F), March (Ma), April (Ap), May (My), June (Ju), July (Jy), August (Au), September (S)

**Table:**
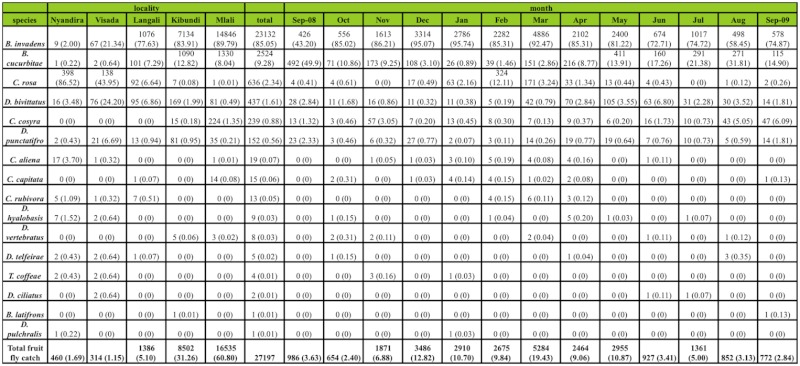
Fruit fly temporal and spatial abundance (percentage of total fruit fly catch).

## References

[bibr01] Aluja M, Guillén L, Rull J, Höhn H, Frey J, Graf B, Samietz J (2011). Is the alpine divide becoming more permeable to biological invasions? Insights on the invasion and establishment of the walnut husk fly, *Rhagoletis completa* (Diptera: Tephritidae) in Switzerland.. *Bulletin of Entomological Research*.

[bibr02] Bale JS, Masters GJ, Hodkinson JD, Awmack C, Bezemer TM, Brown VK, Butterfield J, Chen IC, Shiu HJ, Benedick S, Holloway JD, Cheye VK, Barlow HS, Hill JK, Thomas CD (2009). Elevation increases in moth assemblages over 42 years on a tropical mountain.. *Proceedings of the National Academy of Sciences USA*.

[bibr03] De Meyer M, Copeland RS, Lux SA, Mansell M, Quilici S, Wharton RA, White IM, Zenz N (2002). Annotated checklist of host plants for Afrotropical fruit flies (Diptera: Tephritidae) of the genus *Ceratitis*.. *Documentation Royal Zoological Museum for Central Africa, Tervuren*.

[bibr04] De Meyer M, Robertson MP, Peterson AT, Mansell MW (2008). Ecological niches and potential geographical distributions of Mediterranean fruit fly (*Ceratitis capitata)* and Natal fruit fly (*Ceratitis rosa*).. *Journal of Biogeography*.

[bibr05] De Meyer M, Robertson MP, Mansell MW, Ekesi S, Tsuruta K, Mwaiko W, Vayssières JF, Peterson AT (2010). Ecological niche and potential geographic distribution of the invasive fruit fly *Bactrocera invadens* (Diptera, Tephritidae).. *Bulletin of Entomological Research*.

[bibr06] Dukes JS, Mooney HA (1999). Does global change increase the success of biological invaders?. *Trends in Ecology and Evolution*.

[bibr07] Duyck PF, David P, Quilici S (2004). A review of relationships between interspecific competition and invasions in fruit flies (Diptera: Tephritidae).. *Ecological Entomology*.

[bibr08] Duyck PF, David P, Junod G, Brunei C, Dupont R, Quilici S (2006a). Importance of competition mechanisms in successive invasions by polyphagous tephritids in La Reunion.. *Ecology*.

[bibr09] Duyck PF, David P, Quilici S (2006b). Climatic niche partitioning following successive invasions by fruit flies in La Reunion.. *Journal of Animal Ecology*.

[bibr10] Duyck PF, David P, Quilici S (2007). Can more K-selected species be better invaders? A case study of fruit flies in La Reunion.. *Diversity and Distributions*.

[bibr11] Eggermont H, Verschuren D, Audenaert L, Lens L, Russell J, Klaassen G, Heiri O (2010). Limnological and ecological sensitivity of Rwenzori mountain lakes to climate warming.. *Hydrobiologia*.

[bibr12] Ekesi S, Nderitu PW, Rwomushana I (2006). Field infestation, life history and demographic parameters of the fruit fly *Bactrocera invadens* (Diptera: Tephritidae) in Africa.. *Bulletin of Entomological Research*.

[bibr13] Ekesi S, Nderitu PW, Chang CL (2007). Adaptation to and small-scale rearing of invasive fruit fly *Bactrocera invadens* (Diptera: Tephritidae) on artificial diet.. *Annals of the Entomological Society of America*.

[bibr14] Grout TG, Stoltz KC (2007). Developmental rates at constant temperatures of three economically important *Ceratitis* spp. (Diptera: Tephritidae) from southern Africa.. *Environmental Entomology*.

[bibr15] Hodkinson ID (2005). Terrestrial insects along elevation gradients: species and community responses to altitude.. *Biological Reviews*.

[bibr16] Kottek M, Grieser J, Beck C, Rudolf B, Rubel F (2006). World map of the Koppen-Geiger climate classification updated.. *Meteorologische Zeitschrift*.

[bibr17] Liebhold AM, Johnson DN, Bjornstad ON (2006). Geographic variation in density-dependent dynamics impacts the synchronizing effect of dispersal and regional stochasticity.. *Population Ecology*.

[bibr18] Mwatawala MW, White IM, Maerere AP, Senkondo FJ, De Meyer M (2004). A new invasive *Bactrocera* species (Diptera: Tephritidae) in Tanzania.. *African Entomology*.

[bibr19] Mwatawala MW, De Meyer M, Makundi RH, Maerere AP (2006a). Biodiversity of fruit flies (Diptera, Tephritidae) in orchards in different agro-ecological zones of the Morogoro region, Tanzania.. *Fruits*.

[bibr20] Mwatawala MW, De Meyer M, Makundi RH, Maerere AP (2006b). Seasonality and host utilization of the invasive fruit fly, *Bactrocera invadens* (Dipt., Tephritidae) in central Tanzania.. *Journal of Applied Entomology*.

[bibr21] Mwatawala MW, De Meyer M, Makundi RH, Maerere AP (2009a). An overview of *Bactrocera* (Diptera: Tephritidae) invasions and their speculated dominancy over native fruit fly species in Tanzania.. *Journal of Entomology*.

[bibr22] Mwatawala MW, De Meyer M, Makundi RH, Maerere AP (2009b). Host range and distribution of fruit-infesting pestiferous fruit flies (Diptera, Tephritidae) in selected areas of Central Tanzania.. *Bulletin of Entomological Research*.

[bibr23] Mwatawala M, Maerere AP, Makundi R, De Meyer M (2010). Incidence and host range of the melon fruit fly *Bactrocera cucurbitae* (Coquillett) (Diptera: Tephritidae) in Central Tanzania.. *International Journal of Pest Management*.

[bibr24] R Development Core Team (2008). *R: A language and environment for statistical computing.*.

[bibr25] Rwomushana I, Ekesi S, Gordon I, Ogol C (2008a). Host plants and host plant preference studies for *Bactrocera invadens* (Diptera: Tephritidae) in Kenya, a new invasive fruit fly species in Africa.. *Annals of the Entomological Society of America*.

[bibr26] Rwomushana I, Ekesi S, Ogol C, Gordon I (2008b). Effect of temperature on development and survival of immature stages of *Bactrocera invadens* (Diptera: Tephritidae).. *Journal of Applied Entomology*.

[bibr27] Sakai AK, Allendorf FW, Holt JS, Lodge DM, Molofsky J, With KA, Baughman S, Cabin RJ, Cohen JE, Ellstrand NC, McCauley DE, O'Neil P, Parker EVI, Thompson JN, Weiler SG (2001). The population biology of invasive species.. *Annual Review of Ecology and Systematics*.

[bibr28] Sax DF, Stachowicz JJ, Brown JH, Bruno JF, Dawson MN, Gaines SD, Grosberg RK, Hastings A, Holt RD, Mayfield MM, O'Connor MI, Rice WR (2007). Ecological and evolutionary insights from species invasions.. *Trends in Ecology and Evolution*.

[bibr29] Shelly T, Nishimoto J, Diaz A, Leathers J, War M, Shoemaker R, Al-Zubaidy M, Joseph D (2010). Capture probability of released males of two *Bactrocera* species (Diptera: Tephritidae) in detection traps in California.. *Journal of Economic Entomology*.

[bibr30] ter Braak CJF, Šmilauer P (2002). *CANOCO version 4.5.*.

[bibr31] Theoharides KA, Dukes JS (2007). Plant invasion across space and time: factors affecting nonindigenous species success during four stages of invasion.. *New Phytologist*.

[bibr32] Vargas RI, Shelly TE, Leblanc L, Pinero JC (2010). Recent advances in methyl eugenol and cue-lure Technologies for fruit fly detection, monitoring, and control in Hawaii.. *Vitamins and Hormones*.

[bibr33] Vargas RI, Walsh WA, Kanehisa D, Jang EB, Armstrong JW (1997). Demography of four Hawaiian fruit flies (Diptera: Tephritidae) reared at five constant temperatures.. *Annals of the Entomological Society of America*.

[bibr34] Vayssières JF, Goergen G, Lokossou O, Dossa P, Akponon C (2005). A new *Bactrocera* species in Benin among mango fruit fly (Diptera: Tephritidae) species.. *Fruits*.

[bibr35] Vayssières JF, Carel Y, Coubes M, Duyck PF (2008). Development of immature stages and comparative demography of two cucurbit-attacking fruit flies in Reunion Island: *Bactrocera cucurbitae* and *Dacus ciliatus* (Diptera: Tephritidae).. *Environmental Entomology*.

[bibr36] Vayssières JF, Korie S, Ayegnon D (2009). Correlation of fruit fly (Diptera Tephritidae) infestation of major mango cultivars in Borgou (Benin) with abiotic and biotic factors and assessment of damage.. *Crop Protection*.

[bibr37] Virgilio M, Delatte H, Backeljau T, De Meyer M (2010). Macrogeographic population structuring in the cosmopolitan agricultural pest *Bactrocera cucurbitae* (Diptera: Tephritidae).. *Molecular Ecology*.

[bibr38] Ward NL, Masters GJ (2007). Linking climate change and species invasion: an illustration using insect herbivores.. *Global Change Biology*.

[bibr39] Woodward CA, Shulmeister J (2008). New Zealand chironomids as proxies for human-induced and natural environmental change: Transfer functions for temperature and lake production (chlorophyll a).. *Journal of Paleolimnology*.

[bibr40] Zhang E, Jones R, Bedford A, Langdon P, Tang H (2007). A chironomid-based salinity inference model from lakes on the Tibetan Plateau.. *Journal of Paleolimnology*.

